# Supplementary material for: Radiation-Induced Hypothyroidism in Patients with Oropharyngeal Cancer Treated with IMRT: Independent and External Validation of Five Normal Tissue Complication Probability Models
Source: Cancers (Basel). 2020 Sep 22;12(9):2716. doi: 10.3390/cancers12092716 (PMC7563778; doi:10.3390/cancers12092716)
Supplement: Supplementary file 1 [file cancers-12-02716-s001.zip › cancers-923814-supplementary/cancers-923814-supplementary table and figure.docx]

Article

Radiation-Induced Hypothyroidism in Patients with Oropharyngeal Cancer Treated with IMRT: Independent and External Validation of Five Normal Tissue Complication Probability Models

Zuzanna Nowicka ^1,†^, Bartłomiej Tomasik ^1,2,†^, Anna Papis-Ubych ^3^, Robert Bibik ^4^,
Łukasz Graczyk ^4^, Tomasz Latusek ^5^, Tomasz Rutkowski ^6^, Krystyna Wyka ^7^, Jacek Fijuth ^2^, Jonathan D. Schoenfeld ^8^, Justyna Chałubińska-Fendler ^9^ and Wojciech Fendler ^1,10,^*

**Table 1.** Comparison of patient cohorts from the current study and from the studies developing the evaluated NTCP models.

| **Variable** | **Title** | **Bakhshandeh et al. [14]** | **Boomsma et al. [15]** | **Cella et al. [16]** | **Rønjom et al. [17]** | **Vogelius et al. [18]** | **Present study** |
| --- | --- | --- | --- | --- | --- | --- | --- |
| Number of patients | - | 65 | 105 | 53 | 198 | Meta-analysis of 4 studies (total: 1027 patients) | 108 |
| Number (fraction) developing RIHT |  | 29 (44.6%) | 35 (33%) | 22 (41.5%) | 19 (9.6%) | 342 (33.3%) | 31 (28.7%) |
| Tumour site/diagnosis | - | Nasopharynx (17%)  Oral cavity (15.4%)  Larynx (38.4%)  Other (29.2%) | Oral cavity (14.3%)  Oropharynx (27.6%)  Larynx (32.4%)  Other (25.5%) | Hodgkin lymphoma (100%) | Oral cavity (3.0%)  Oropharynx (50.5%)  Hypopharynx (5.1%)  Larynx (41.4%) | Head and neck cancer (51.9%), Hodgkin lymphoma (48.1%) | Oropharynx (100%) |
| Planned radiation dose [Gy] | - | Range: 46-66 | Range: 46-66 | Range: 30-36 | Range: 60-68 | Range: 30-70 | 70 |
| Sex | Male | 41 (63.1%) | 28 (26.7%) | 25 (47.2%) | 155 (78.3%) | Not reported | 84 (77.8 %) |
|  | Female | 24 (36.9%) | 77 (73.3%) | 28 (52.8%) | 43 (21.7%) |  | 24 (22.2 %) |
| Age [years] | - | Median: 54  (range: 28-86) | 18-49 years: 16.2%  50-59 years: 24.8%  60-69 years: 30.5%  ≥70 years: 28.6% | Median: 27.5 (range: 14-70) | Median: 61.2 (range: 31.4-85.0) | Range: 3-82 years | Median: 60 (range: 35-77) |
| Concomitant chemotherapy | Yes | 33 (50.8%) | 15 (14.4%) | 53 (100%) | 78 (39.4%) | Not reported | 65 (60.2%) |
|  | No | 32 (49.2%) | 82 (85.6%) | 0 (0%) | 120 (60.6%) |  | 43 (39.8%) |
| Surgery | Yes | 32 (49.2%) | Not reported | Not reported | 61 (30.8%) | Not reported | 6 (5.6 %) |
|  | No | 33 (50.8%) |  |  | 137 (69.2%) |  | 102 (94.4 %) |
| Time to follow-up | - | <12 months after RT | Median: 2.5 years after RT | Median: 32 months after RT | Median: 22.4 months after RT | 1-11 years after RT | Median: 28 months after RT |
| Mean thyroid dose [Gy] | - | Median: 42.2  (range: 13-65) | Not reported | Not reported | Median: 33.5 (range: 3.8-64.8) | Not reported | Median: 52.7 (range: 15.0-69.2) |
| Thyroid volume [cm3] | - | 14.5 ± 8.3 | Not reported | Not reported | Median: 17.3 (range: 6.4-85) | Not reported | Median: 19.0 (range: 1.8-77.6) |
| Thyroid V30 | - | Not reported | Not reported | Not reported | Not reported | Not reported | Median: 100.0 (range: 24.6-100) |
| Baseline TSH [mIU/L] | - | 1.7 ± 1.2 | Not reported | Not reported | Median: 0.9 (range: 0.3-3.4) | Not reported | Median: 0.7 (range: 0.03-2.47) |

**Table 2.** Parameters of logistic function describing the recalibrated NTCP models.

| **Parameter** | **Bakhshandeh et al. [14]** | **Boomsma et al. [15]** | **Cella et al. [16]** | **Rønjom et al. [17]** | **Vogelius et al. [18]** |
| --- | --- | --- | --- | --- | --- |
| Intercept | -4.01 | -5.65 | -18.22 | -4.00 | -4.02 |
| x | 5.20 | 8.47 | 17.31 | 6.63 | 4.73 |
| Performance | | | | | |
| Brier score | 0.197 | 0.106 | 0.216 | 0.102 | 0.203 |
| Nagelkerke R^2^ | 0.093 | 0.500 | 0.086 | 0.585 | 0.106 |


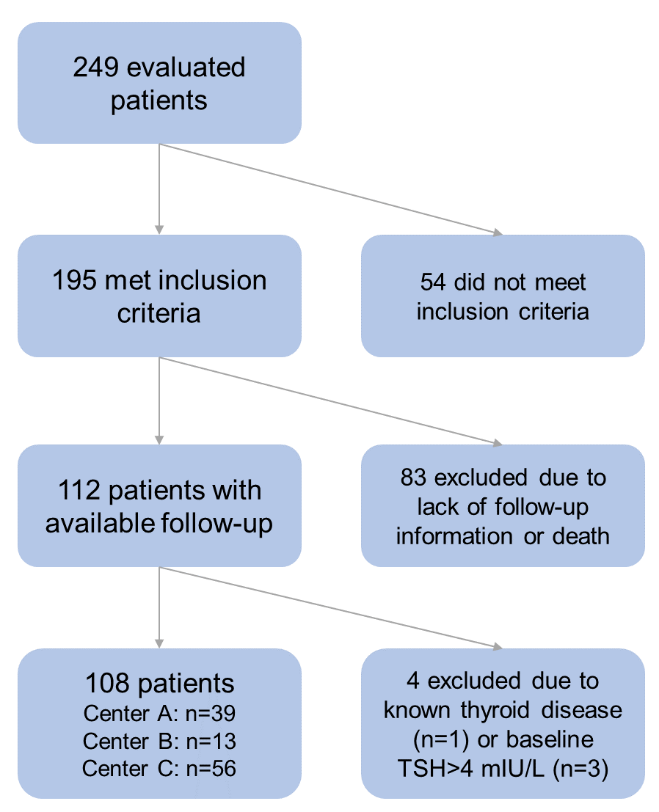


**Figure 1.** Diagram of patient flow through the study.


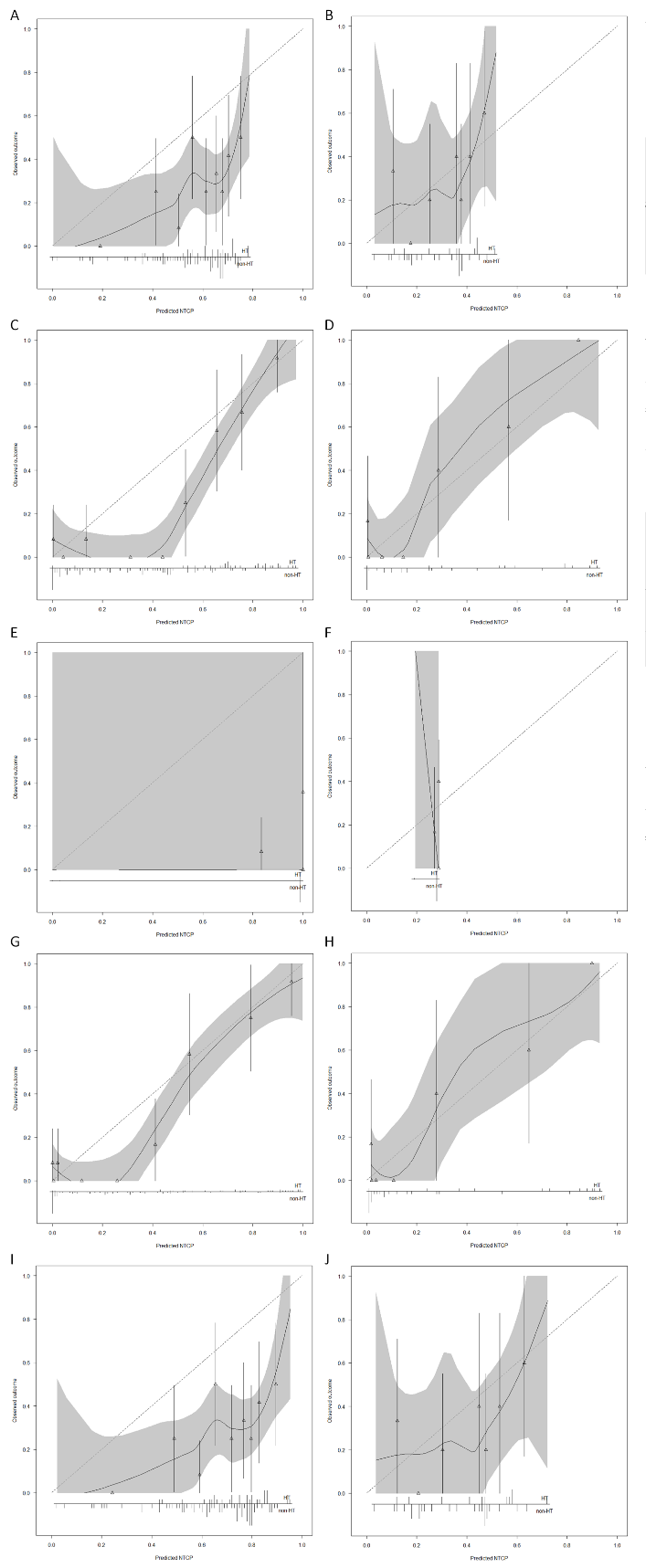


**Figure 2.** Calibration plots for all original and recalibrated NTCP models, respectively: (A) and (B) by Bakhshandeh et al [14]., (C) and (D) by Boomsma et al [15]., (E) and (F) by Cella et al [16]., (G) and (H) by Rønjom et al [17]., (I) and (J) by Vogelius et al [18].
